# MechanoAge, a machine learning platform to identify individuals susceptible to breast cancer based on mechanical properties of single cells

**DOI:** 10.1016/j.ebiom.2026.106241

**Published:** 2026-04-23

**Authors:** Stefan Hinz, Sturla M. Grøndal, Masaru Miyano, Jennifer C. Lopez, Kristen L. Cotner, Taylor Thomsen, Chang Chen, Edward J. Hester, Lisa D. Yee, Victoria E. Seewaldt, James B. Lorens, Lydia L. Sohn, Mark A. LaBarge

**Affiliations:** a Department of Population Sciences, Beckman Research Institute, City of Hope, Duarte, CA, USA; b Department of Biomedicine & Centre for Cancer Biomarkers, University of Bergen, Bergen, Norway; c UC Berkeley–UC San Francisco Graduate Program in Bioengineering, University of California, Berkeley, CA, USA; d Department of Mechanical Engineering, University of California, Berkeley, CA, USA; e Department of Surgery, City of Hope Comprehensive Cancer Center, Duarte, CA, USA; f Center for Cancer and Aging Research, City of Hope, Duarte, CA, USA

**Keywords:** Single-cell mechanical phenotyping, Breast cancer risk, Keratin-14 remodelling, Microfluidic node-pore sensing, Machine learning

## Abstract

**Background:**

Emerging evidence links cellular ageing and biophysical alterations with cancer susceptibility. Existing breast cancer risk models inadequately identify individuals at latent risk, particularly among women without known genetic mutations or family history. Risk is often underestimated or overestimated due to reliance on population-level data and absence of individualised tissue-based markers of breast cancer risk.

**Methods:**

We profiled primary human mammary epithelial cells (HMECs) from women of varying ages and risk backgrounds using mechano-node-pore sensing (mechano-NPS), a high-throughput microfluidic platform that measures single-cell physical and mechanical properties. We developed a machine learning classifier, MechanoAge, to estimate chronological age based on mechanical phenotypes, and a biological age-based risk index, Mechano-RISQ. We further assessed cytoskeletal protein keratin 14 (KRT14) as a key mediator of underlying mechanical states through overexpression and knockdown experiments.

**Findings:**

Epithelial cells from normal tissue of young BRCA1/2 mutation carriers (n = 4), women with family history of breast cancer (n = 3), and tissue contralateral to a tumour-bearing breast (n = 9) exhibited elevated Mechano-RISQ scores, which reflects accelerated biological ageing compared to age-matched controls (n = 18). KRT14 overexpression induced a biologically aged phenotype in cells obtained from younger women, whereas knockdown partially reversed this state in cells from older women. CyTOF profiling and modelling showed KRT14 modulation impacted protein expression signatures associated with ageing and risk.

**Interpretation:**

Mechano-RISQ offers a proof of principle approach for identifying individuals at elevated risk of breast cancer, especially among average-risk populations, and may complement existing risk models by incorporating biophysical measures of mammary epithelial cell ageing.

**Funding:**

NIH R01EB024989, R01CA237602, and P30CA033572, DOD BC181737, American Cancer Society—Fred Ross Desert Spirit Postdoctoral Fellowship (PF-21-184-01-CSM).

**Research in context**

**Evidence before this study**

Breast cancer risk increases with age, and women carrying germline variants such as BRCA1, BRCA2, or PALB2 face substantially elevated lifetime risk. Accumulating evidence indicates that ageing does not accelerate carcinogenesis but instead alters cell-intrinsic and microenvironmental states, increasing susceptibility to cancer initiation. Prior work has shown that mammary epithelia from young women with high-risk germline variants exhibit accelerated biological ageing, including changes in intermediate filament organisation, molecular ageing clocks, and stromal immune features. However, the functional consequences of these ageing-associated changes, and whether they could be detected in normal tissue from women without known inherited pathogenic gene variants, remain unclear.

**Added value of this study**

We show that physical and mechanical properties of normal human mammary epithelial cells provide a functional readout of biological age and breast cancer susceptibility. Using mechano-node-pore sensing and machine learning, we define a mechanical age (MechanoAge) and a deviation metric (Mechano-RISQ) that quantify age-discordant mechanical states at single-cell resolution. Normal epithelial cells from women with germline mutations, strong family history of cancer, or contralateral breast cancer exhibit mechanically aged phenotypes despite normal histology. Modulation of KRT14 is sufficient to shift mechanical age in both directions, identifying a cytoskeletal mechanism that links ageing biology to emergent mechanical states associated with risk.

**Implications of all the available evidence**

Together with prior molecular and epigenetic studies, these findings support a model in which accelerated biological ageing of mammary epithelia may underpin breast cancer susceptibility across genetic and non-genetic risk groups. Mechanical phenotyping captures an integrative cellular state that reflects underlying molecular networks rather than single biomarkers. This approach could enable earlier, individualised risk stratification, particularly for women who lack identifiable high-risk mutations yet harbour susceptible tissue states.

## Introduction

Breast cancer, as one of the most frequently diagnosed cancers worldwide and a leading cause of cancer-related mortality among women, has long been the subject of efforts to improve risk stratification and early-detection strategies. Despite considerable advances in both screening technologies and therapeutic interventions, accurately determining which individuals, particularly among those considered average-risk, are most likely to develop the disease remains one of the most persistent challenges in oncology and public health.

Current risk models such as the Gail and Tyrer-Cuzick attempt to integrate clinical and demographic variables including age, reproductive history, family history, and benign breast disease to estimate breast cancer risk over defined periods.^1^ Although widely used in clinical practice these models suffer from known limitations: the Gail model tends to underestimate risk in many women, while the Tyrer-Cuzick model overestimates risk, especially in those with atypical hyperplasia upon biopsy.^2^ More importantly, both models rely on population-level data and offer limited insight into an individual's ongoing biological responses to ageing or environmental exposures. In particular, these empirical estimates fall short in accounting for how biological processes in breast cells affect an individual's underlying susceptibility to the disease.

Genetic testing for high-penetrance mutations such as those in BRCA1 and BRCA2 has yielded powerful predictive tools for the small subset of women whose risk is hereditary, which are estimated to comprise just 5–10% of all cases. Women harbouring these mutations may face a lifetime breast cancer risk as high as 83%,^3^^,^^4^ and first-degree family history without known germline risk alleles confers a lifetime risk of approximately 21%.^5^ Yet a majority of breast cancers arise in women with no family history and no known pathogenic variants, and whose estimated lifetime risk is 13%. Among ostensibly average-risk individuals, it remains difficult to identify those with latent risk that stems from cellular, molecular, and biophysical alterations that current models are not designed to capture.

This gap is especially concerning in the context of early-onset breast cancer, where incidence has been rising at an alarming rate. Between 2012 and 2021, breast cancer incidence increased by 1.4% annually in women under 50, compared to 0.7% in older women, and was nearly twice that rate in certain ancestry groups.^6^ These trends suggest that ancestry-related biology and non-hereditary contributors play a larger role than previously appreciated. Environmental exposures, lifestyle changes, delayed childbearing, and rising prevalence of obesity are all potential contributing factors, yet we still lack a predictive framework that incorporates how these external variables interact with internal biological ageing processes in the mammary epithelium.

To address this need, we propose that changes in the mechanical properties of cells, which are long recognised as hallmarks of both ageing and disease,^7^ offer a previously unexamined opportunity to assess individual susceptibility to breast cancer. Ageing and disease progression are associated with alterations in cellular elasticity, stiffness, and morphology, which stem from cytoskeletal remodelling, matrix interactions, and disrupted mechanotransduction pathways. These mechanical changes are functional reflections of the underlying cell state and contain discriminative information capable of resolving subtle differences in lineage commitment, chronological age, and malignant potential.

In previous work, we showed that genome- and proteome-wide changes in human mammary epithelial cells (HMECs) with age correlate with high-risk phenotypes, and that the cytoskeletal intermediate filament protein keratin 14 (KRT14) is differentially expressed in luminal epithelial cells of older women.^8^ Importantly, hallmarks that we associated with ageing, including this key cytoskeletal change, are accelerated by decades in young women who carry BRCA1, BRCA2, or PALB2 germline mutations compared to average risk.^9^^,^^10^ These observations suggest that biophysical phenotyping may serve as a surrogate for biological ageing and cancer risk, particularly in HMECs. To quantify these properties at single-cell resolution and in high throughput, we employed mechano-node-pore sensing (mechano-NPS), a microfluidic platform that yields large, multi-parametric datasets that capture mechanical features of individual cells.^11^^,^^12^

Here, using mechano-NPS, we generated profiles of physical and mechanical properties of primary HMECs from donors of varying ages and cancer risk levels and applied machine learning (ML) to classify those phenotypes as a function of age in average risk women. The phenotypes were predictive of breast cancer susceptibility and associated with accelerated biological ageing, as measured mechanically, in young women with germline mutations in BRCA1 or BRCA2, or with strong family histories of breast cancer, or with cancers in the contralateral breast. We introduce a new index, mechano-Risk Index for Single-cell Quantification (Mechano-RISQ), to describe this biophysical signature of cancer-prone ageing epithelia. Furthermore, we investigated the role of KRT14 in modulating this phenotype and found this cytoskeletal keratin integral to the mechanical state of high-risk cells.

While other ageing biomarkers such as epigenetic clocks or telomere length provide valuable perspectives, they capture only slices of the complex and multilayered biology of ageing. By contrast, mechanical phenotyping, anchored in cytoskeletal dynamics and cell–matrix interactions, represents an integrative, systems-level perspective that directly reflects cellular function and risk state. As such, we propose Mechano-RISQ as a innovative class of biomarker that fills a longstanding void in risk prediction: identifying individuals with accelerated ageing-driven susceptibility to breast cancer before malignancy emerges.

## Methods

### Primary epithelial cells

**Fig. 1:**
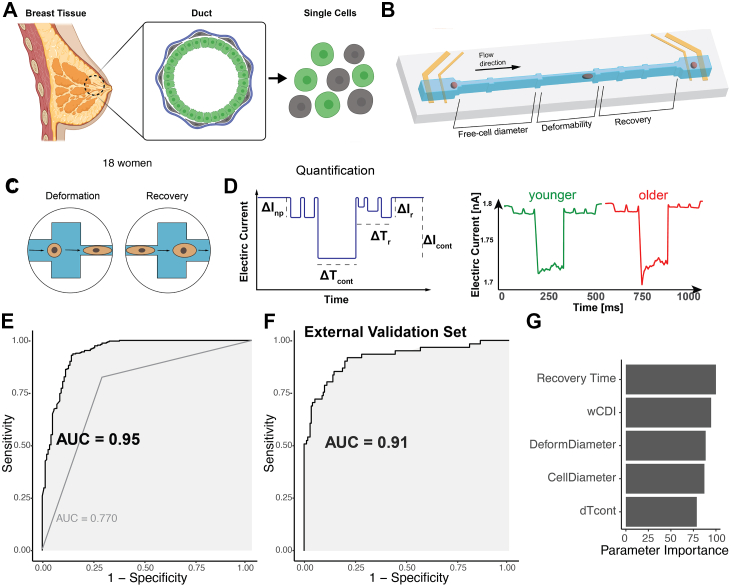
**Age-dependent mechanobiological profiles in breast cancer**. (A) Schematic of breast tissue structure and cell isolation. (B) Overview of the mechano-NPS microfluidic device used to measure single-cell mechanical properties. (C) Example cell deformation and recovery process within the mechano-NPS device. (D) Expected current pulse caused by a cell transiting the device. The pulse consists of subpulses reflecting the three main regions of the channel. Cell size $\left(D_{\text{cell}}\right)$ is determined by the magnitude of the initial subpulse $\left(\Delta I_{\text{np}}\right)$, where $\Delta I_{\text{np}}/I_{\text{baseline}}∼V_{\text{cell}}/V_{c\text{hannel}}$ are the cell and channel volumes, respectively). Cell stiffness is determined by the transit time of a cell passing through the contraction channel $\left(\Delta T_{\text{cont}}\right)$; stiffer cells take longer than softer ones to transit through. To normalise with respect to cell size, we define the whole cell deformability index (*wCDI*) as $\left(L_{c}/U_{\text{flow}}h_{\mathrm{channel}}\right)\left(D_{\text{cell}}/∆T_{\mathrm{cont}}\right)$, where $L_{\text{cont}}$ is the length of the contraction channel, $U_{\text{flow}}$ is the velocity of the fluid, and $h_{\text{channel}}$ is the channel height. *wCDI* is inversely related to Young's modulus. Recovery time $\left(\Delta T_{r}\right)$ is defined as the time needed for the magnitude of the subpulses after the contraction channel $\left(\Delta I_{r}\right)$ to return to $\Delta I_{\text{np}}$. Representative pulses of electric current, illustrating current drops associated with cell deformation and recovery within the microfluidic channel. (E) ROC curve illustrating the performance of MechanoAge's classification of age groups based on validation data (18 women). (F) ROC curve illustrating the performance of MechanoAge's classification of age groups based on validation data (5 women) (G) Feature importance derived from the mechanobiological classifier, highlighting the top five mechanical parameters contributing to the risk prediction.

Primary HMEC strains were derived from leftover breast tissues from reduction mammoplasty and prophylactic mastectomy specimens (Fig. 1A) and were propagated for four passages. We have shown that epithelial phenotypes in this system undergo minimal adaptation from uncultured organoids through passage four, which mitigates concerns that the mechanophenotypes reported here arise from culture adaptations.^10^^,^^13^ Primary HMEC strains were maintained as described previously.^14^^,^^15^ The HMEC strains used in this study are listed in Supplementary Table S1. All cultures were screened regularly and remained mycoplasma-free throughout the study. For lineage-specific mechanical measurements, cells were separated using Stem Cell's EasySep technology and stained with CD271 and CD133 antibodies. For transduction studies, HMECs were transduced with lentiviral particles in the presence of polybrene (10 μg/mL) and were selected using puromycin (2 μg/mL) or hygromycin (10 μg/mL).

### Mechano-NPS device design

The mechano-NPS devices used in this study consisted of a 22.3 μm-high polydimethylsiloxane (PDMS) microfluidic channel bonded to a glass substrate with pre-defined platinum (Pt) electrodes and gold (Au) contact pads. The channel consists of a series of nodes and pores on either side of a single “contraction channel.” Each node is 50 μm × 85 μm (L × W), each pore is 700 μm × 22 μm (L × W), and the contraction channel is 3000 μm × 10.5 μm (L × W). The contraction channel width, *w*_c_, was chosen to apply an average strain, *ε*_avg_ = (*D*_cell-avg_−*w*_c_)/*D*_cell-avg_ ∼ 0.4, over a sufficient amount of time to each cell transiting the microfluidic device (Supplementary Table S2). Here, *D*_cell_ is the free cell diameter. The recovery length segment is 700 μm long.

### Mechano-NPS Device Fabrication

Mechano-NPS devices were fabricated, as previously published.^11^^,^^12^^,^^16^ Briefly, to create the PDMS microfluidic moulds, standard photolithography was first used to fabricate negative-relief masters on polished silicon wafers. PDMS (Sylgard 184, Dow Corning), mixed at a ratio of 10:1 pre-polymer: curing agent and degassed, was then poured onto the negative-relief masters and cured at 85 °C for 2 h. A slab of PDMS with the embedded microfluidic channel was excised from the master, and input and outlet ports were cored with a 1.5 mm diameter biopsy punch. Completion of the mechano-NPS device involved exposing a PDMS mould and a glass substrate with pre-defined electrodes to an oxygen plasma (Harrick Plasma, 450 mTorr (60 Pa), 30 W, 2 min) and aligning and mating the two together. Permanent bonding of the glass substrate and PDMS mould was accomplished by heating the device on a hotplate at 85 °C for 2 h. To fabricate the Pt electrodes and Au contact pads onto the glass substrates, standard photolithography was used for patterning. A trilayer of thin metal film (50/250/250 Å titanium/Pt/Au) was deposited via electron-gun evaporation and a subsequent lift-off with acetone was performed. Gold wet etch (Gold Etchant TFA, Transene Company) was used to expose the Pt electrodes.

### Mechano-NPS device measurement

Single-cell suspensions of HMECs (∼2 × 10^5^ cells/mL) were resuspended in PBS and immediately injected into the device. A constant DC voltage (1 V) was applied across the channel, and a four-terminal measurement of the current was used to measure the modulated current pulses caused cells transiting the channel when a pressure of 25 kPA was used. We employed custom-written software (see Zenodo https://doi.org/10.5281/zenodo.14927530) to extract both the magnitude and duration of each current sub-pulse (Δ*I*_np_, Δ*I*_c_, Δ*T*_cont_, and Δ*T*_r_). Δ*I*_np_ corresponds to the current drop as the cell enters the node-pore region at the beginning of the device, Δ*I*_c_ is the current drop as the cell enters the contraction channel, Δ*T*_cont_ is the cell's transit time through the contraction channel, and Δ*T*_r_ is the time necessary for the cell to relax to its original shape after deformation (Fig. 1D). If a cell remains deformed past the observation period (120 ms for the device geometry and applied flow rate employed), its relaxation time is recorded as “Inf.”

### Determination of the free cell diameter, *D*_cell_, transverse deformation, recovery time, and whole cell deformability index, *wCDI*

The magnitude of the current sub-pulse, Δ*I*_*np*_/*I*, produced in the node-pores prior to the contraction channel provides information on the *D*_cell_. Specifically,

(1) $$\frac{\Delta I}{I}=\frac{D_{cell}^{3}}{D_{e}^{2}L}\left[\frac{1}{1-0.8{\left(D_{cell}/D_{e}\right)}^{3}}\right]$$

where *D*_e_ and *L* are the effective diameter and length of the channel, respectively. See Ref.^11^ for more details on the derivation of Eq. (1). *D*_e_ is determined by measuring polystyrene microspheres of known size with the microfluidic channel. Once *D*_e_ is known, then *D*_cell_ of a screened cell can be numerically solved in Eq. (1) using the obtained values of Δ*I*_np_/*I*.

To determine a cell's transverse deformation,^11^ we assume that the cell is an oblate spheroid when in the contraction channel. Its volume is *V*_deform_ = π*w*_c_(*L*_deform_)^2^/6 where *L*_deform_ is the cell's deformation length. As the magnitude of the current change in the contraction channel, Δ*I*_c_/*I*, is proportional to the volume ratio of the deformed cell and contraction channel, *V*_deform_/*V*_contraction_, the transverse deformation of the cell is thus *δ*_deform_ = *L*_deform_/*D*_cell_.

To determine the recovery time of a cell after applied strain, Δ*T*_r_, we note the time required for the sub-pulses produced by the cell *after* exiting the contraction channel to return to the same shape and magnitude as those produced by the cell *prior* to entering the contraction channel. For the device dimensions used in this study, the temporal window is 120 ms for the applied flow rate.

The *wCDI* is a dimensionless parameter, which was previously shown to be inversely related to cellular cortical tension or Young's modulus.^11^ This parameter is defined as,

(2) $$wCDI=\frac{L_{C}}{U_{flow}h_{channel}}\;\frac{D_{cell}}{{\Delta T}_{cont}}$$

where *U*_flow_ is the fluid velocity in the node section leading into the contraction channel, *L*_c_ is the length of the contraction channel, and *h*_channel_ is the contraction-channel height. *U*_flow_, *L*_c_, and *h*_channel_ are fixed values for any given experiment, and consequently, *D*_cell_ and Δ*T*_*cont*_ are the key parameters in the *wCDI*. As noted in Kim et al. (2018)^11^ the *wCDI* describes the deformability of the cell as a whole, including the cytoskeleton, nucleus, and organelles. Those cells that are more deformable, transit the contraction channel more easily and correspondingly at higher velocities, resulting in a higher *wCDI*. Notably, the *wCDI* negates cell-size effects, as cells that are larger (smaller) will transit the contraction channel more slowly (quickly).

### Classifying age groups using mechano-NPS data

A database of mechano-NPS measurements was created from HMECs of 18 women, including 1381 cells in the training set and 661 cells in the validation dataset. Cells were manually classified based on chronological age into two groups: “older” (>50 years of age) and “younger” (<35 years of age). The modelling was performed in the R programming environment using the caret package. A pseudocode representation of this workflow is included in Algorithm 1 and Zenodo https://doi.org/10.5281/zenodo.18776439. Dummy Variables were derived from the non-continuous “RecoveryTime” variable to represent five distinct recovery time intervals (binary indicators for recovery time bins: 0 ms; 50–60 ms; 60–70 ms; 70–120 ms; and infinite recovery). In total the model used ten cell-level mechanophenotype features. A Yeo-Johnson transformation was applied to improve the normality of the data distribution and ensure variance homogeneity. Three ML algorithms were employed to model the relationship between predictor variables and age categories: Bagged Decision Trees,^17^ Random Forest,^18^ and Extremely Randomised Trees,^19^ all implemented using the caret package.^20^ Bagged decision trees were trained using 50 bootstrap resamples without additional hyperparameter tuning. Random forest models were optimised using caret's automated tuning procedure (tuneLength = 30) to select the number of variables randomly sampled at each split. Extremely randomised trees were trained using default tuning parameters, leveraging the algorithm's inherent randomisation to reduce variance. Model training was performed using repeated k-fold cross-validation (10 folds, five repeats) to ensure robust performance estimation optimised for ROC AUC. A down-sampling strategy was employed to address minor class imbalance in the outcome variable. An ensemble model was constructed using the caretStack function from the caretEnsemble package^21^ to enhance predictive performance. The ensemble combined the predictions of the three individual models using a Generalised Boosted Regression Model (GBM)^22^ as the meta-learner. The GBM was configured with a tuning length of 10, and model training was performed using 5-fold cross-validation. The performance metric was set to ROC AUC, and a down-sampling strategy was maintained to address class imbalance. Down-sampling was applied only to the training data during model fitting to address class imbalance. Specifically, the majority class was randomly down-sampled within each resampling fold defined by the cross-validation procedure implemented in caret. The test and validation datasets were not downsampled to ensure unbiased performance evaluation. The final optimised ensemble model was applied to the validation and independent validation dataset to generate class predictions and associated probabilities. Variable importance was calculated using caret::varImp() for each base learner and summarised by averaging importance scores across models to obtain a consensus ranking.

**Algorithm 1:**
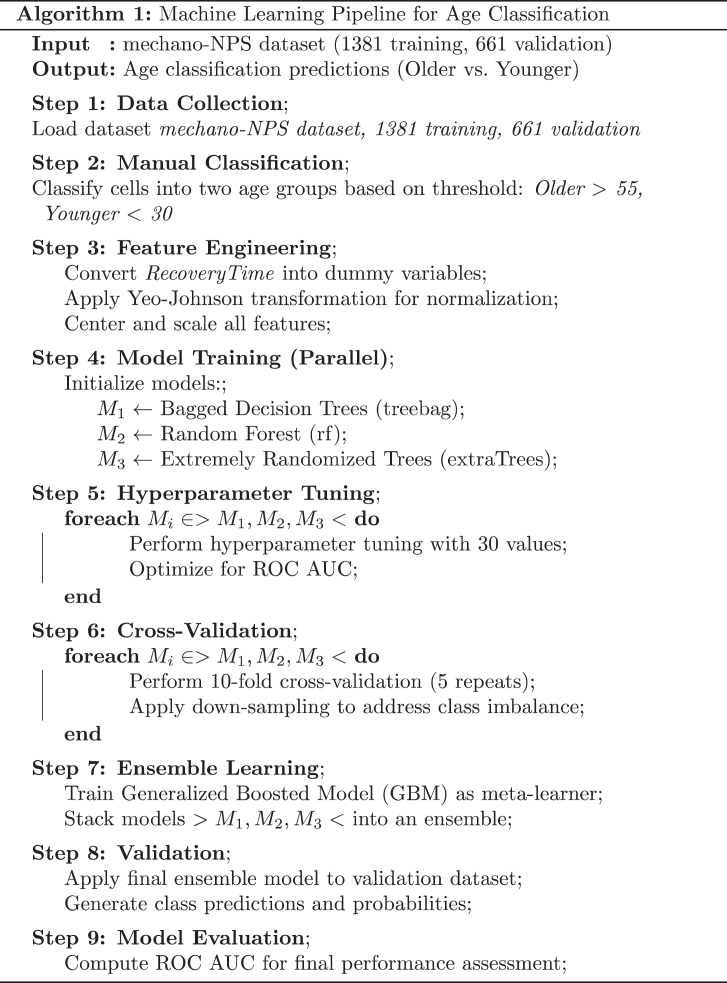
Pseudocode for MechanoAge.

### Mechano-Risk Index for Single-cell quantification (RISQ) score calculation

The Mechano-RISQ score quantifies the deviation of cell classification as “older” relative to a defined baseline error rate in the context of mechanical and age-related phenotype predictions. The baseline error rate represents the proportion of cells misclassified as “older” in the core model, determined from average risk HMECs. This value was used as a reference to calculate the relative increase or decrease in the proportion of misclassified cells across experimental conditions. To calculate the Mechano-RISQ score for each experimental condition, the percentage of misclassified cells was compared to the baseline error rate to compute the ratio of deviation. A ratio of 1.0 indicates no deviation from the baseline, while values above or below 1.0 reflect an increase or decrease in the misclassification rate, respectively.

### CyTOF

Pre-conjugated antibodies were obtained from Standard Biotools (San Francisco, USA) (Supplementary Table S3). Unlabelled antibodies were conjugated in-house using the Maxpar® ×8 Antibody Labelling Kit (Standard BioTools) or the MIBItag Conjugation Kit (Ionpath, Menlo Park, CA, USA). Primary HMECs were stained for viability with cisplatin according to the manufacturer's protocol and then fixed with paraformaldehyde (PFA). Samples were incubated in PBS before primary barcoding into three separate pools using the. After barcoding, the combined pool was aliquoted for titration, validation, and acquisition. Titration samples were used to optimise marker concentrations, validation samples confirmed titration accuracy, and acquisition samples were reserved for final data collection. The combined barcode pool washed and stained with a mixture of metal-conjugated antibodies against surface proteins (Supplementary Table S3). After staining, cells were washed in CSB, fixed in 2% PFA, and permeabilised in methanol. Subsequently, cells were washed in CSB and stained with a cocktail of metal-conjugated antibodies for intracellular proteins (Supplementary Table S3). Following staining, cells were fixed in 2% PFA with Cell-ID™ Intercalator-Ir (Standard BioTools). For acquisition cells were diluted to 0.1× and acquired using the tuned Helios mass cytometer with the Super Sampler. Gating strategy for mass cytometry shown in Supplementary Figure S1. Differentially expressed proteins (DEPs) between groups were analysed on a per-protein basis, applying a non-parametric Wilcoxon rank-sum test to detect significant differences in expression. Multiple hypothesis testing corrections were applied using the Bonferroni method, adjusting *P*-values. Proteins were reported as significant if they met predefined thresholds of adjusted *P*-value <0.05 and |LFC| > 0.5.

### CyTOF expression signatures

CyTOF differential protein signatures were modelled using gradient-boosting machines. Separate binary classifiers were trained for each biological comparison (younger vs older and younger vs high-risk) using luminal epithelial cell protein features. For each comparison, models were trained once using unperturbed control samples with a stratified 70/30 train–test split and five-fold cross-validation to maximise AUC. The trained models were subsequently applied, without retraining, to KRT14 overexpression, shKRT14 knockdown, and combined perturbation conditions, and ROC curves (sensitivity vs 1-specificity) were used to evaluate classification performance across experimental settings.

### Statistics

All statistical analyses were performed in the R programming environment. Group comparisons of Mechano-RISQ scores were performed using the two-sided Wilcoxon rank-sum test, a non-parametric test chosen because mechano-NPS measurements are not assumed to follow a normal distribution and sample sizes per donor are unequal. For CyTOF differential protein expression analyses, per-protein differences between groups were assessed using the Wilcoxon rank-sum test with Bonferroni correction for multiple hypothesis testing; proteins were considered significant at adjusted *P* < 0.05 and |log fold change| > 0.5. Differences in the proportion of cells classified as “older” between KRT14 perturbation conditions and controls were assessed using Fisher's exact test, appropriate given the binary classification outcome and small cell counts in some strata. Classification performance of MechanoAge was evaluated using receiver operating characteristic (ROC) analysis, with area under the curve (AUC) reported as the primary performance metric; 95% confidence intervals for AUC were computed using the DeLong method. Single-case Mechano-RISQ values were compared against the normative average-risk distribution using the Crawford–Howell t-test, a method validated for single-case vs normative group comparisons; power for this test was estimated at >0.97 via simulation (10,000 iterations, Cohen's d = 3, α = 0.05).

Formal a priori sample size determination was not performed, as there are no established conventions for power calculations in this class of single-cell mechanical phenotyping studies. Study size was instead justified empirically through a learning curve analysis in which random subsamples of 40–1300 cells were drawn five times each; the observed plateau in ROC AUC and reduction in standard error with increasing sample size indicated that the current training database of 1381 cells from 18 donors approached the model's optimal performance regime (Supplemental Figure S2).

Down-sampling of the majority class was applied within each cross-validation fold during model training to address minor class imbalance; test and validation datasets were not down-sampled to ensure unbiased performance evaluation.

Mechano-NPS measurements are instrument-derived and do not require operator interpretation.

Inclusion criteria for HMEC strains were: derivation from normal breast tissue, donor chronological age either >50 years (older group) or <35 years (younger group). High-risk and family history samples were not included in model training and were used exclusively for independent evaluation of Mechano-RISQ.

### Ethics

All primary human mammary epithelial cell (HMEC) strains used in this study were derived from de-identified, discarded surgical tissue obtained under an honest broker arrangement, in accordance with institutional policy. Tissue was collected under IRB protocol IRB17185, approved by the Institutional Review Board at City of Hope. All patients provided written informed consent prior to surgery under this tissue collection protocol. The study exclusively used normal breast tissue; no samples were obtained directly from breast cancer lesions. Donors included women undergoing prophylactic or contralateral mastectomy due to understood elevated risk for future breast cancer, and women undergoing cosmetic reduction mammoplasty.

### Role of funders

The funders had no role in study design; data collection, analysis, or interpretation; or in the writing of the manuscript.

## Results

### Database creation

We used primary HMECs from 18 women of varying ages (Supplemental Table S1) as a model system to investigate ageing-related changes (Fig. 1A). HMECs offer a unique advantage because they can be obtained from multiple samples, allowing the analysis of a diverse range of specimens. Unlike immortalised cell lines, primary HMECs maintain genomic stability and acquire minimal tissue culture adaptations, providing a biologically relevant system for examining age-associated cellular changes processes by comparing cells obtained from women of different chronological ages.^10^^,^^15^ We measured the mechanical properties of the cells via mechano-NPS.^11^ This platform consists of a microfluidic channel segmented by larger areas called “nodes”; one segment between two nodes, the “contraction channel”, is narrower than the diameter of a cell, causing the cell to experience an applied constant strain of *ε*_*avg*_ ∼ 0.4 (Supplementary Table S2) in these studies for a defined period (Fig. 1B and C). A four-terminal measurement of the current across the microfluidic channel measured the modulating current caused by a transiting cell. An analysis of the modulated current provided information on cell diameter, stiffness, and recovery from deformation^11^ (Fig. 1D, see Methods and Supplementary Information).^11^ By leveraging primary HMECs in conjunction with high-resolution mechanical data, we quantified mechanical changes across a broad age spectrum. We created a training dataset comprising over 1500 single-cell mechanical profiles from 18 women.

### Model performance

We modelled the relationship between cellular mechanical features and age using ensemble ML algorithms, including Bagged Decision Trees, Random Forests, and Extremely Randomised Trees. These algorithms are well-suited for biological data as they effectively capture non-linear interactions, handle high-dimensional datasets, and are robust against noise, predictor correlations, and the variability inherent in biological measurements.^23^ Our model — MechanoAge — predicts the age category of HMECs at the single-cell level based on mechanical phenotypes measured using the mechano-NPS platform. MechanoAge has a high predictive accuracy: the Receiver Operating Characteristic (ROC) curve, with an area under the curve (AUC) of 0.95 (Fig. 1E), indicates MechanoAge's exceptional ability to distinguish between different age categories of HMECs. This performance assessment was based solely on validation data that was not used during training. The ROC curve indicated a strong balance between sensitivity and specificity across various thresholds and ensured accurate classification of HMECs into younger or older categories. This robustness across thresholds suggested that MechanoAge consistently predicted accurately, across a wide range of cut-off points.

Additional validation was performed using an independent external sample cohort (n_cells_ = 281, n_sample_ = 5). ROC analysis on this dataset yielded an AUC of 0.91 (Fig. 1F), supporting the robustness of MechanoAge performance on data from previously unseen samples. To further assess the robustness of model performance to individual sampling, we computed sample-level learning curves across 50 random sample inclusion orders, each evaluated using leave-one-sample-out validation. Model performance improved rapidly with increasing sample number and stabilised after approximately 8–10 samples, whereas variability across sample orderings decreased (Supplementary Figure S3). These results indicate that the observed performance is not driven by a small subset of samples and that the current cohort size is sufficient to support the reported sample-level trends in this proof-of-concept study. Despite an increase trend, the proportion of luminal epithelial cells in HMEC primary cultures did not differ significantly between age groups, indicating comparable epithelial lineage composition across the biobank. This suggests that MechanoAge discrimination reflects age-associated mechanical changes within epithelial cells rather than differences in luminal–myoepithelial cell proportions. Thus, MechanoAge was sensitive to true mechanobiological age-related signals and remained resistant to variations in the test data. Fig. 1G illustrates the importance of the top five mechanical properties measured by mechano-NPS platform in predicting the age of HMECs. “Recovery Time” emerged as the most critical feature and indicated that the time it took for a cell to recover after deformation was a key determinant in classifying its age category. For training purposes, Recovery Time was divided into five distinct binary variables; the cumulative contribution of these variables is shown in Fig. 1G. This was closely followed by the “wCDI” (whole-cell deformability index), a dimensionless parameter a analytically derived by Kim et al. (2018) that has been experimentally shown to be inversely related to cellular cortical tension and elastic modulus (see Methods), “Deform Diameter” (the transverse deformation of the cell under constant strain in mechano-NPS contraction channel), and “Cell Diameter” (the “free” diameter of the cells), all of which significantly enhanced the MechanoAge's predictive power. We trained a model excluding cell diameter and deformability-related diameter features. This reduced performance modestly compared with the full feature set (AUC = 0.823; ΔAUC = −0.09), indicating that while size-related features contribute to classification, the model is not driven by cell size. A lesser importance was attributed to “ΔTcont” (the cell's transit time through the contraction channel).

When only considering the fraction of slow-recovering cells after deformation, as previously observed by Kim et al. (2018) AUC-, the property showed a striking decrease in performance, with an AUC of 0.77 (Fig. 1E). Whereas Recovery Time was a significant individual predictor of age, the lower AUC compared to that from MechanoAge (AUC of >0.91) indicated that this parameter alone did not capture the full complexity of the data. The reduced AUC signified a diminished capability to classify accurately cells into their corresponding age categories because the additional mechanical characteristics provided complementary information. These data imply that the various mechanical properties measured by the mechano-NPS platform interacted in critical ways for differentiating between age categories, such that relying on a single feature led to a significant loss of information.

### Mechanical age as a predictor of risk

Age is the principal risk factor for developing breast cancer.^24^ We previously showed that some biochemical and transcriptomic breast-specific ageing hallmarks were accelerated in women who harboured pathogenic alleles of BRCA1, BRCA2, or PALB2.^13^^,^^25^ Given the ability of our MechanoAge model to predict accurately the age category based on mechanical characteristics of mammary epithelial cells, we determined whether mechano-NPS could detect aberrations in “mechano-age,” which would then serve as early indicators of elevated breast cancer risk in women with high-risk mutations.

**Fig. 2:**
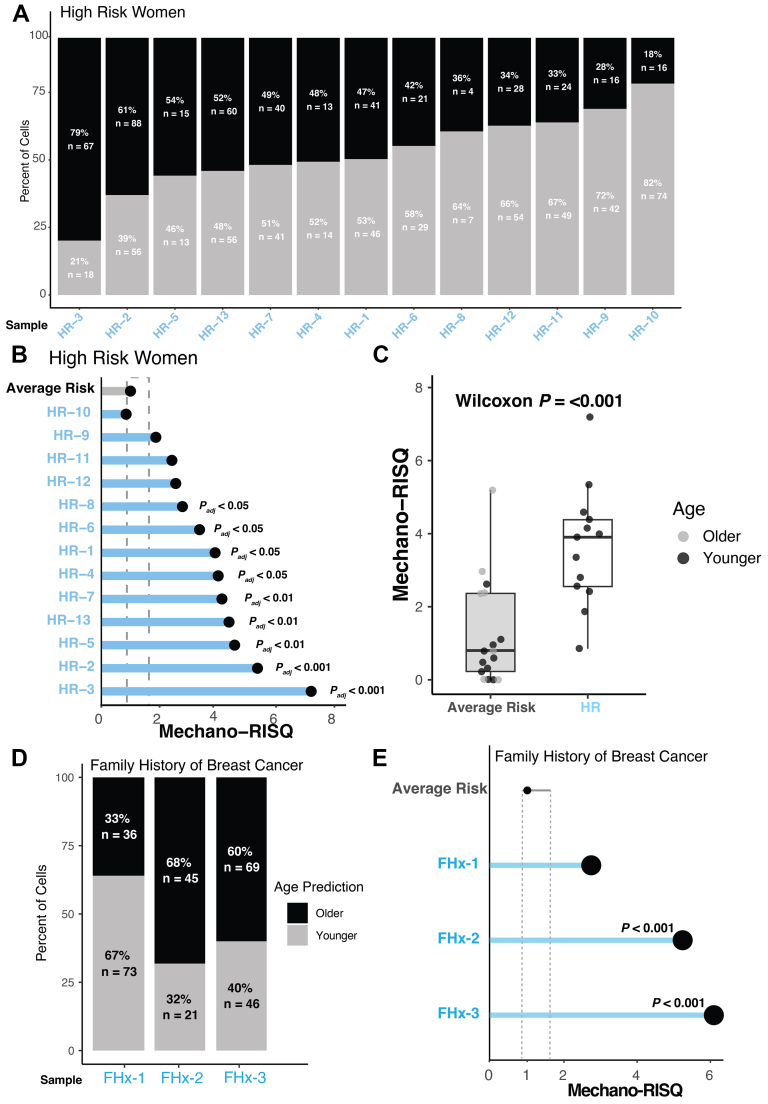
**Age-linked mechanobiological profiles enhance risk stratification in breast cancer.** (A) Bar plots showing the percentage of cell age classification based on their mechanobiological signatures in high-risk (HR) patients. n indicates cells per sample. (B) Dot plot illustrating the Mechano-RISQ in individuals with high-risk factors for developing breast cancer. Significance was determined using Crawford–Howell test after Benjamini-Hochberg *P* value correction per sample. Dotted line: 95% confidence interval. (C) Grouped Mechano-RISQ comparison between average risk and high risk individuals. (D) Stacked bar plots showing the percentage of cell age classification in individuals with a family history of breast cancer. n indicates cells per sample. (E) Dot plot demonstrating the Mechano-RISQ for individuals with a family history of breast cancer. Significance was determined using Crawford–Howell test. Dotted line: 95% confidence interval.

We applied MechanoAge to HMECs from normal tissue of women with anticipated elevated breast cancer risk, including: carriers of pathogenic BRCA1/2 alleles, individuals with contralateral breast cancers (ductal carcinoma in situ (DCIS) or invasive breast cancer (IBC) in the other breast), and women with a family history of breast cancer and no recognised high-risk alleles. Across the high-risk cohort (HR-1 to HR-13), a substantial fraction of cells from chronologically younger donors were classified as “older” based on their mechanophenotype (Fig. 2A). This effect was most pronounced in BRCA2 carriers (HR-1, HR-3) and BRCA1 carriers (HR-2, HR-13), where 47–79% of cells exhibited an older age prediction despite donor ages under 35 years. Although not a dominant factor in MechanoAge, we observed that cell diameters of HR cells trended smaller than average risk (Supplemental Table S2). Similar age-shifted physical and mechanical phenotypes were also observed in non-germline high-risk conditions, including tissue contralateral to breast cancer, indicating that the underlying biology of susceptibility extends beyond inherited BRCA mutations.

To quantify deviations from expected age classification, we developed the Mechano-RISQ score, an odds-ratio–inspired metric comparing observed age misclassification to that expected for average-risk samples. Mechano-RISQ scores were consistently elevated across high-risk samples relative to average-risk controls (Fig. 2B). HR samples showed significantly increased Mechano-RISQ values. At the group level, high-risk samples exhibited significantly higher Mechano-RISQ scores than average-risk samples (Wilcoxon *P* < 0.001; Fig. 2C), indicating a systematic shift toward an older mechanobiological state.^10^ We next evaluated cells from women with a family history (FHx) of breast cancer without any identified HR mutations (Fig. 2D), a group for whom the assessment of true risk was more challenging due to the absence of definitive genetic markers. Younger FHx individuals exhibited a higher proportion of cells classified as “older” than expected. The cells isolated from younger FHx individuals exhibited significantly elevated Mechano-RISQ scores compared to the average-risk population (*P* < 0.001 for FHx-2, FHx-3), which indicated an aged mechanical phenotype (Fig. 2E). Thus, cells from these individuals with a family history of breast cancer, even in the absence of detected known high-risk alleles, remarkably had emergent mechanical properties shared with cells known to be HR.

Together, these data indicate that accelerated mechanobiological ageing is a shared feature across genetically defined and clinically defined breast cancer risk states. The convergence of elevated Mechano-RISQ scores across BRCA1/2 carriers, contralateral to carcinoma samples, and FHx samples supports the idea that breast cancer risk is accompanied by emergent mechanical properties at the single-cell level that precede overt disease. This pattern parallels prior observations from an orthogonal molecular assay in which germline high-risk mutations advanced biological age estimates derived from an epigenomic ELF5 clock,^10^ reinforcing the concept that elevated breast cancer risk is associated with precocious ageing signatures in the mammary epithelium.

### Keratin 14 as a molecular mediator of age-associated mechanical states

**Fig. 3:**
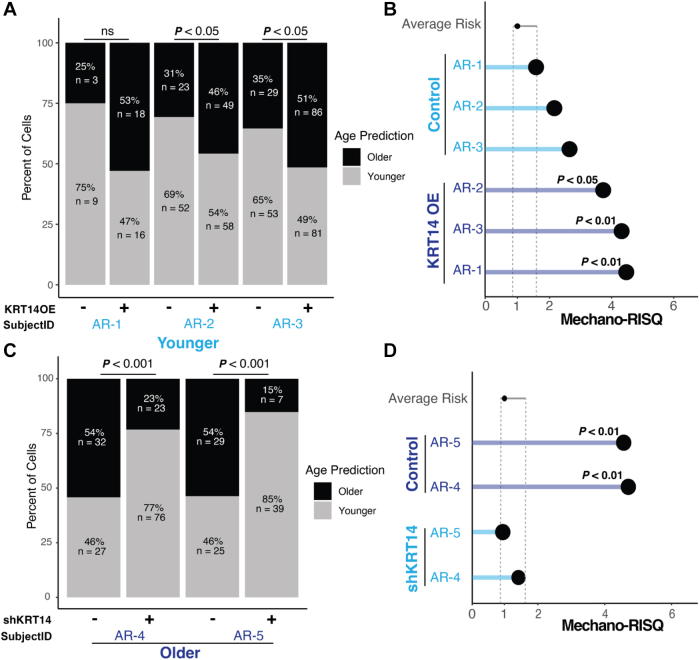
**Modulation of Keratin 14 alters predicted cellular age and Mechano-risk score**. (A) Bar plots showing the percentage of cells predicted as older or younger based on the age prediction MechanoAge across KRT14 overexpression (KRT14OE) and control conditions in luminal cells. Significance was determined using Fisher's exact test. n indicates cells per sample. (B) Dot plot showing Mechano-RISQ scores for younger cells under KRT14OE and control conditions in luminal cells. Statistical significance is shown for changes in risk scores per sample. Significance was determined using the Crawford–Howell test. Dotted line: 95% confidence interval of testing dataset. (C) Bar plots showing the percentage of cells predicted as older or younger across shKRT14 and control conditions in older luminal cells. Significance was determined using Fisher's exact test. n indicates cells per sample. (D) Dot plot showing Mechano-RISQ scores for older cells under shKRT14 and control conditions in luminal cells. Statistical significance is shown for changes in risk scores per sample. Significance was determined using the Crawford–Howell test. Dotted line: 95% confidence interval.

To investigate the molecular underpinnings of the observed mechanical changes and their effect on age predictions, we focused on KRT14, an intermediate filament and marker of myoepithelial cells in younger mammary epithelia. KRT14 upregulation occurs with increasing age and in luminal cells of BRCA1/2, PALB2 gene mutation carriers.^8^^,^^26^^,^^27^ Given KRT14's role in cytoskeleton, we explored how its overexpression and knockdown influenced mechanobiological properties, specifically regarding age prediction accuracy. We examined the effects of KRT14 overexpression in luminal HMECs from three average-risk (AR) younger women (AR-1, AR-2, and AR-3). KRT14 overexpression increased the proportion of cells classified as older in all three strains, with significant differences in AR-2 and AR-3 (*P* < 0.05, Fisher's exact test); and AR-1 displayed a similar trend (Fig. 3A). KRT14 overexpression in the HMECs resulted in significantly elevated Mechano-RISQ scores (*P* < 0.05 and *P* < 0.01, respectively), increasing the probability of younger cells being classified as older (Fig. 3B).

Next, we examined the effects of KRT14 knockdown (shKRT14) in older luminal cells and focused on two individuals (AR-4 and AR-5). KRT14 knockdown led to a significant reduction in the proportion of cells classified as “older” in both (*P* < 0.001) (Fig. 3C) and significantly reduced Mechano-RISQ (Fig. 3D). In AR-5, only 15% of cells were classified as older following KRT14 knockdown, compared to 85% classified as younger. A similar distribution was observed in AR-4, where the proportion of cells classified as older dropped to 23%, which indicated that KRT14 knockdown in older cells substantially reduced older mechanical phenotype. This suggested a critical role of KRT14 in the mechanical ageing process, and that its knockdown partially reversed the age-related biophysical changes in older cells.

KRT14, a key cytoskeletal protein, significantly influenced the mechanical properties of younger and older cells. Overexpression of KRT14 in younger cells promoted an “older” mechanical phenotype, whereas the knock down of KRT14 in older cells reduced their likelihood of being classified as older. This highlights KRT14's central role in age-associated mechanical states.

### KRT14 modulates molecular signatures and mechanical phenotypes

**Fig. 4:**
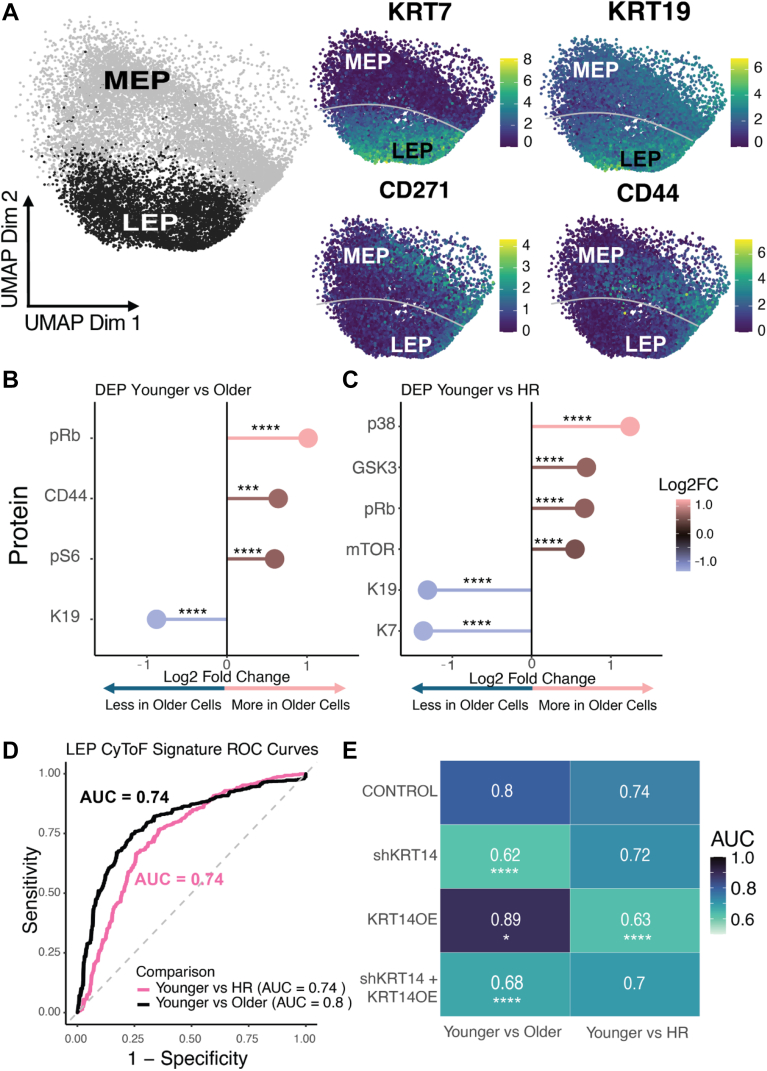
**CyTOF analysis of luminal epithelial cells across age and risk groups**. (A) UMAP visualisation of luminal epithelial (LEP) and myoepithelial (MEP) cell populations with protein expression mapped for selected markers. (B) Differential protein expression in luminal epithelial cells between younger and older groups and (C) younger vs HR groups. (D) ROC curves generated from GBM models based on differential protein expression, demonstrating predictive capacity for age and high-risk status. Five-fold cross-validation was performed at the single-cell level. ROC/AUC and statistical testing were based on single-cell prediction scores. (E) Heatmap of AUC values comparing experimental conditions in younger vs older and high-risk groups. Significance was determined using DeLong's test and compared to control. Dotted line: 95% confidence interval. Five-fold cross-validation was performed at the single-cell level. ROC/AUC and statistical testing were based on single-cell prediction scores.

To investigate age- and risk-associated protein expression associated with mechanical states, we analysed ∼14,000 HMECs from three younger AR, three older AR, and six HR individuals. Using CyTOF, we assessed 27 proteins and phospho-proteins relevant to cell-cycle regulation, signalling, and cytoskeletal dynamics (Supplemental Table S3). The panel was adapted from a previously validated framework (Pelissier-Vatter et al.),^26^ augmented with additional markers implicated in cytoskeletal regulation. UMAP visualisation of control HMECs (Fig. 4A) showed distinct aggregation of luminal and myoepithelial populations, with notable variability in protein expression for known lineage markers such as KRT7, KRT19, CD271, and CD44.

Differential protein abundance analysis revealed significant shifts across age and risk groups. Comparisons between younger and older individuals (Fig. 4B) identified increased expression of pRb, CD44, and pS6 and decreased levels of KRT19 in older cells, which indicated age-associated changes in cell-cycle regulation, signalling, and cytoskeletal integrity. Similarly, comparisons between younger and HR strains (Fig. 4C) highlighted the altered expression of p38, GSK3, and mTOR.

**Fig. 5:**
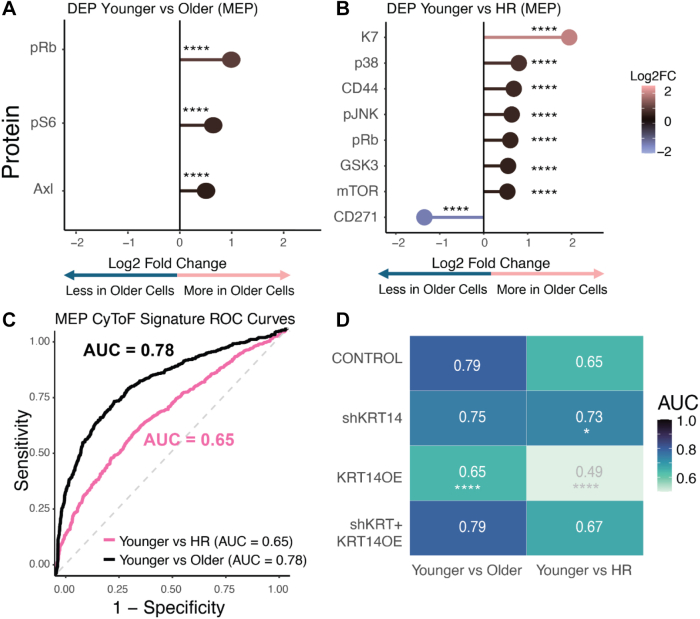
**CyTOF analysis of myoepithelial cells across age and risk groups**. (A) Differential protein expression in myoepithelial (MEP) cells between younger vs older and (B) younger vs HR groups. (C) ROC curves generated from GBM models based on differential protein expression, demonstrating predictive capacity for age and high-risk status. Five-fold cross-validation was performed at the single-cell level. ROC/AUC and statistical testing were based on single-cell prediction scores. (D) Heatmap of AUC values comparing experimental conditions in younger vs older and high-risk groups. Significance was determined using DeLong's test and compared to control condition. Five-fold cross-validation was performed at the single-cell level. ROC/AUC and statistical testing were based on single-cell prediction scores. Significance is indicated as follows: ∗*P* < 0.05; ∗∗*P* < 0.01; ∗∗∗*P* < 0.001, ∗∗∗∗*P* < 0.0001.

We performed KRT14 overexpression (OE), KRT14 knockdown (shKRT14), and dual modulation (shKRT14 + KRT14OE) experiments to examine how gene expression alterations influence protein signatures in HMECs. The “KRT14OE” construct was designed such that the shKRT14 short hairpin RNA could not target the KRT14 mRNA that was overexpressed, which enabled simultaneous overexpression and knockdown in the same cells and only ablated the autologous KRT14 expression in the double transduction sample (Supplementary Figure S4). Gradient boosting machine (GBM) models were trained on differentially expressed proteins and produced robust predictive accuracy. GBM algorithms built an ensemble of decision trees sequentially and optimised a loss function through gradient descent (Fig. 4D). For binary classification tasks such as “younger vs older” or “younger vs high-risk,” GBM minimised the log-loss function, similar to logistic regression, to output probabilities for classification per single cell. Unlike logistic regression, GBM did not assume a linear relationship between input variables and the classification variable, which allowed it to capture nonlinear interactions. ROC curves (Fig. 4D) demonstrated strong performance in distinguishing younger vs older (AUC = 0.80). This approach highlighted a separation between average risk younger and HR cells (AUC = 0.74). KRT14 OE had a pronounced impact on the age model, which significantly enhanced predictive capacity (AUC = 0.89), while its effects on the HR model were more modest. Similarly, shKRT14 reduced accuracy predominantly in the age model, with a smaller effect on the HR model (Fig. 4E, Supplementary Figure S5). However, in myoepithelial cells, which naturally exhibited higher KRT14 expression levels, the impact of KRT14 overexpression on the age model was different. The model's ability to distinguish between younger and HR cells was less effective in myoepithelial cells as compared to in luminal cells. Myoepithelial cells exhibited a lower AUC of 0.65 for the same comparison, suggesting a weaker separation, which implied that high-risk signatures may be more pronounced in luminal epithelial cells (Fig. 5, Supplementary Figure S5). Together, these findings suggested that KRT14 modulation caused substantial molecular changes associated with cellular ageing, as evidenced by significant shifts in Mechano-RISQ scores. The risk-associated changes in the high-risk model demonstrated that additional pathways are engaged following KRT14 modulation, indicating that age prediction may be influenced by a broader mechanobiological landscape.

## Discussion

Physical and mechanical phenotypes of single cells offer an underexplored approach to identifying age-associated phenotypes in mammary epithelia that are relevant to breast cancer susceptibility. Accelerated biological ageing features relative to chronologic age are associated with higher risk of breast and other cancers.^28^ This study of mammary epithelial cells obtained from women with varying risk levels and ages offers a much-needed method for refining breast cancer risk assessment at an individual, breast tissue-based level. High-resolution, single-cell physical and mechanical profiling revealed that ageing and HR conditions, such as BRCA1/2 mutations and family history of breast cancer, are associated with distinct mechanical states. That normal tissue contralateral to breast tumours exhibited elevated Mechano-RISQ crucially indicated that the biology underlying cancer susceptibility is not confined to the tumour site but reflects a broader, individual-level state. The ability of MechanoAge to classify cells accurately by age (AUC = 0.91–0.95) and to detect deviations in mechanical phenotypes in individuals with high cancer risk suggests that cellular mechanical properties may be developed as an early biomarker for cancer susceptibility and, thus, as a tool for cancer risk assessment.

The identification of HR individuals for breast cancer remains a significant challenge, particularly in cases where genetic mutations are absent or ambiguous. BRCA1 and BRCA2 mutations account for 5–10% of hereditary breast cancer cases; however, the majority of individuals (>90%) diagnosed with breast cancer lack these mutations.^29^ Moreover, many fall into intermediate-risk categories, such as those with variants of unknown significance (VUS), a family history of breast cancer without identifiable mutations, or presumably influenced by the environmental exposome.^24^ Traditional risk assessment tools often fail to provide precise or actionable insights for these individuals. Our findings suggest that mechanical profiling may address these challenges by detecting emergent subtle, and telling, risk-associated cellular changes that are otherwise undetectable. For instance, mechanical changes occur in HR groups even in the absence of known genetic mutations, as demonstrated by the elevated Mechano-RISQ scores in both BRCA1/2 mutation carriers and in individuals with a family history of breast cancer or a contralateral finding of breast cancer. Similarly, younger individuals with a family history of breast cancer exhibited a higher proportion of cells classified as “older,” further supporting the hypothesis that biophysical alterations may precede detectable genomic changes that increase cancer susceptibility.

A key strength of this study is the use of physical and mechanical phenotyping for uncovering early indicators of susceptibility in populations that are difficult to stratify with current approaches such as germline DNA sequencing, histopathology, and immunohistochemistry. Mechano-NPS is particularly suited for mechanical phenotyping at scale, given its throughput, which is substantially higher than the gold standards of atomic force microscopy and micropipette aspiration.^11^^,^^12^ The integration of multidimensional mechanical data in combination with ensemble ML algorithms offers distinct advantages in this context by providing high-resolution, single-cell mechanical profiles in a high-throughput format. ML was essential in uncovering the combinations of physical and mechanical properties that differentiate otherwise normal HMEC by their age or risk groups. Dissecting the factors that contributed to MechanoAge predictions revealed cell size as an important, though not dominant feature, with high-risk epithelial cells trending ∼0.5 μm smaller than average-risk cells–consistent with engagement of cell-size regulatory pathways. Indeed, YAP1, a key component of Hippo signalling, is dysregulated in luminal epithelial cells with age, and we demonstrated that protracted moderate-level YAP1 activity in ageing and high-risk mammary epithelia drives conditionally immortal cell states that are primed for immortal transformation.^30^ Measurement of mechanical states could serve as a complement to genetic, epigenetic, and lifestyle risk factors to deliver a more comprehensive and individualised risk assessment for breast cancer.

Our investigation into the role of the intermediate filament KRT14 highlights its role in mediating cytoskeletal reorganisation during cellular ageing. Changes in KRT14 expression in mammary epithelia was among the first overt hallmarks of ageing that we observed.^8^^,^^25^ Overexpression of KRT14 in younger cells induced an “older” mechanical phenotype, and knockdown in older cells attenuated age-associated mechanical changes. These findings indicate that KRT14 is a central molecular mediator of cytoskeletal remodelling in this context. The effect of KRT14 modulation on mechanical properties corresponds with distinct protein expression changes. GBM models trained on differentially expressed proteins confirmed the importance of KRT14 in distinguishing cells by age (AUC = 0.80) and younger vs high-risk cells (AUC = 0.74). Whereas KRT14 overexpression strongly enhanced age prediction accuracy (AUC = 0.89), its effects on high-risk classification were less pronounced, suggesting that additional pathways contribute to cancer susceptibility beyond KRT14-driven mechanical alterations.^10^ We noted that perturbation of KRT14 was technically challenging, with HMECs exhibiting reduced cellular fitness following both overexpression and knockdown. This sensitivity implies strong selective pressure to maintain KRT14 levels within a narrow range and supports roles beyond filament mechanics alone. The interactions with adhesion and mechanotransduction pathways, including PEAK1^27^ and Hippo,^30^^,^^31^ as potential nodes connecting cytoskeletal state to cell fitness. The observed changes in protein expression resulting from keratin changes align with the hypothesised role of intermediate filaments as signalling scaffolds.^32^ Consistent with prior work showing that germline HR mutations advance biological age estimates derived from an ELF5-based epigenomic clock,^10^ future studies testing whether KRT14 perturbation similarly shifts ELF5-derived ageing metrics would help link mechanical and transcriptional ageing programs.

Here, we demonstrated that intermediate filaments are key regulators of these biomechanical profiles (Figs. 3 and 4); KRT7,^33^ KRT19,^34^ KRT14^35^ are intermediate filaments, linked by prior research to cancer and cancer risk. Since all cells contain intermediate filaments, these findings likely extend beyond HMECs, and beyond keratins intermediate filaments, suggesting broader relevance across various cell types and tissues. Age-related biomechanical changes may represent a fundamental hallmark of cellular function, with distinct mechanical phenotypes underlying critical processes in ageing, cancer, and potentially other diseases. Recognising and utilising these biomechanical markers could greatly enhance early detection, refine risk stratification, and improve targeted intervention strategies. This could be especially significant for those with ambiguous risk profiles, including individuals without known genetic mutations. Critically, incorporating mechanical phenotyping into clinical trials could enhance trial design by allowing earlier, quantifiable endpoints, particularly in prevention studies, which would facilitate a more accurate evaluation of intervention efficacy and risk reduction strategies.

### Limitations

Several limitations of this study warrant consideration. The cohort size was modest, which necessarily limits power to resolve the full range of inter-individual heterogeneity in breast epithelial ageing and cancer susceptibility. While the signals we observe are internally consistent and biologically coherent, larger and more diverse cohorts will be required to define population-level distributions, refine risk thresholds, and test performance across subgroups.

Mechanical phenotyping was performed on cells measured *ex vivo*. Although we restricted analyses to early-passage primary mammary epithelial cells, tissue dissociation, cryopreservation, and short-term culture may alter aspects of cell state relative to native tissue. These constraints are shared by most single-cell functional assays, but they underscore the importance of validating Mechano-RISQ signatures in freshly isolated clinical samples. Ongoing and future studies using fine-needle aspiration–derived cells measured with minimal processing will be essential to establish robustness under clinically realistic conditions.

The demographic composition of the cohort also represents a limitation. Ancestral diversity was restricted, and it remains unresolved whether the age- and risk-associated mechanical phenotypes described here generalise across populations with different genetic, environmental, and social exposures. Similarly, the role of hormones and metabolic states that might relate to menopause and obesity are not clear in the Mechano-RISQ context. However, based on our extensive prior work these factors do not exert detectable effects on other biomarkers of breast ageing, including KRT14. Expanding both the biological and demographic breadth of sampled individuals will be critical, particularly given known disparities in breast cancer incidence and outcomes.

With respect to the mechano-NPS platform itself, current throughput is approximately 300 cells per minute, which is lower than that achieved by some image-based mechanophenotyping approaches such as hydrodynamic stretching cytometry and real-time deformability cytometry.^36^, ^37^, ^38^, ^39^ That trade-off reflects design choices. Mechano-NPS does not require high-speed imaging, specialised optics, or computationally intensive image analysis, making it inherently scalable. Multiplexed channel designs allow simultaneous measurement of multiple cells and mechanical conditions, enabling future iterations to reach throughputs in the thousands of cells per sample and positioning the platform for clinical screening applications.

In its present configuration, mechano-NPS applies a constant strain during cell transit. This captures a reproducible snapshot of cellular deformability and recovery, but cells are nonlinear viscoelastic materials whose behaviour depends on both strain magnitude and loading frequency.^40^ Modifications to channel geometry, including sinusoidal contraction channels and frequency-tuned designs, would allow measurement of additional parameters such as elasticity and viscosity.^41^ Applying a range of average strains across devices would further expand the mechanical feature space accessible to analysis. Incorporating these dimensions is likely to yield a more complete physical portrait of epithelial state and improve the resolution of MechanoAge models.

Taken together, these limitations define a clear path forward rather than fundamental barriers. Larger, more diverse cohorts, minimally processed clinical samples, and expanded mechanical feature sets will be required to fully establish the generalisability and translational potential of mechanical ageing as a biomarker of breast cancer susceptibility.

## Contributors

Conceptualisation: SH, MM, LLS, and MAL

Writing—Original Draft: SH and MAL.

Writing—Review & Editing: SH, MM, SMG, LLS, JBL, and MAL.

Investigation: SH, MM, SMG, and JCL.

Device Fabrication: KLC, TT, CC, and EJH.

Formal Analysis: SH, SMG.

Data Curation: SH and SMG.

Visualisation: SH.

Biospecimen Provision: VES.

All authors read and approved the final version of the manuscript. SH and MAL accessed and verified the underlying data.

## Data sharing statement

Available upon request, subject to a Material Transfer Agreement (MTA). Contact mlabarge@coh.org.

CyTOF data is deposited through 10.5281/zenodo.15004690.

Model training script is deposited through 10.5281/zenodo.18776439.

## Declaration of interests

LLS is an awardee of U.S. Patent No. 11,383,241: “Mechano-node-pore sensing,” with J. Kim, S. Han, and L. L. Sohn, issued 12 July 2022. Provisional patent application US62/830,328 has been submitted by S. Hinz, M. A. LaBarge, and L. L. Sohn. The authors declare that they have no further competing interests.

## Appendix A Supplementary data

Supplementary Figures and Tables

Supplemental Western Blots
