# Supplementary material for: MechanoAge, a machine learning platform to identify individuals susceptible to breast cancer based on mechanical properties of single cells
Source: eBioMedicine. 2026 Apr 23;127:106241. doi: 10.1016/j.ebiom.2026.106241 (PMC13174242; doi:10.1016/j.ebiom.2026.106241)
Supplement: Supplementary Figures and Tables [file mmc1.docx]

## Supplemental Figures


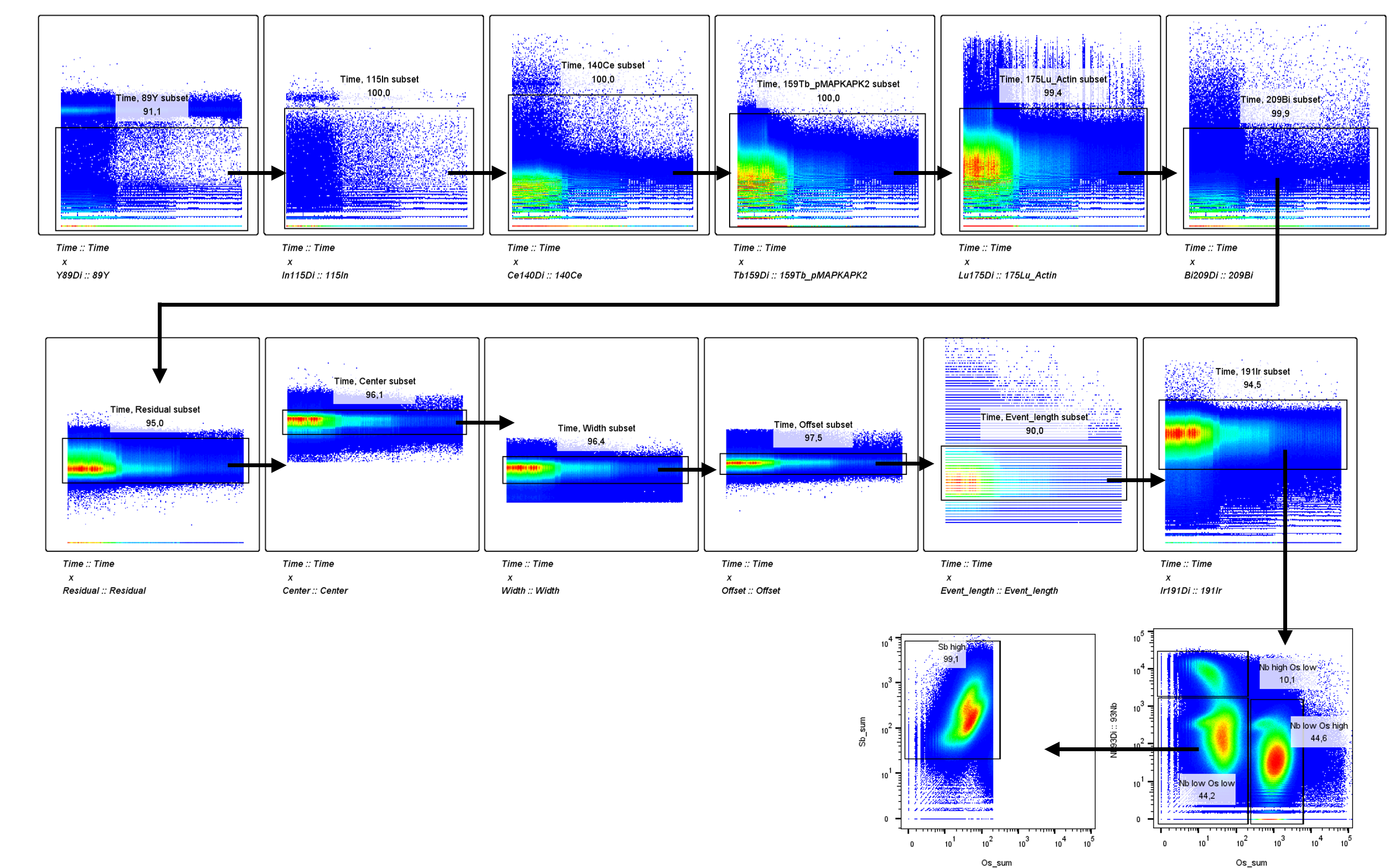


**Supplemental Figure 1: Gating strategy for mass cytometry data. Gating was performed on normalised and concatenated events.**

Top row of gate on beads for every channel. Second row applies gating on gaussian parameters and DNA (191Ir). Third row gates out barcode pools based on staining of niobium, osmium, and antimony.

##
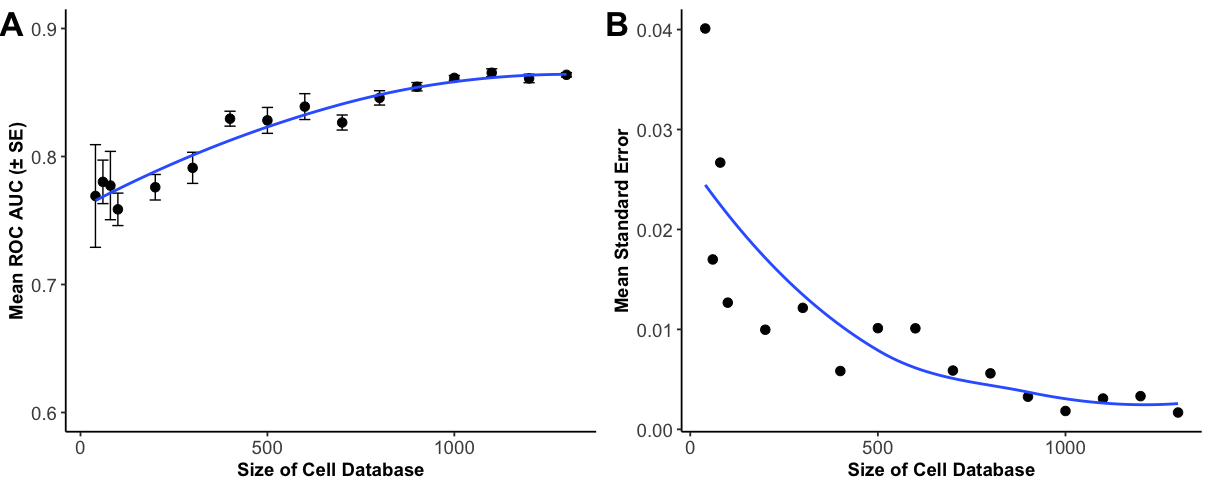


**Supplemental Figure 2: Learning Curve Analysis for Estimating Cell Database Size Requirements:** (A) Mean ROC AUC (± SE) plotted against the size of the cell database. (B) Mean standard error of the AUC for the same samples.

**Supplemental Figure 3: Sample-level learning curve.**

Median area under the ROC curve (AUC) as a function of the number of samples included, evaluated using leave-one-out validation of the full ensemble model. For each sample count, performance was computed across 50 random sample addition orders, each starting with one younger and one older sample. The solid line shows the median AUC, and the shaded region indicates the interquartile range (25th–75th percentile). Base learners and the stacking model were refit within each sample subset to prevent sample-level information leakage.


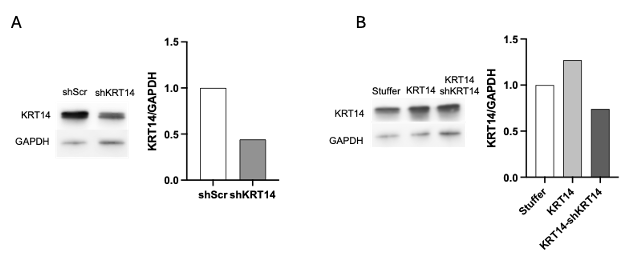


**Supplementary Figure 4: Validation of KRT14 knockdown and overexpression at the protein level.** (A) Representative western blot showing reduced KRT14 protein levels in shKRT14 cells compared to scrambled control (shScr). (B) Representative western blot showing increased KRT14 protein levels in KRT14-overexpressing cells relative to stuffer controls, with partial reduction upon co-expression with shKRT14. GAPDH served as a loading control. Bar graphs show densitometric quantification of KRT14 normalised to GAPDH.


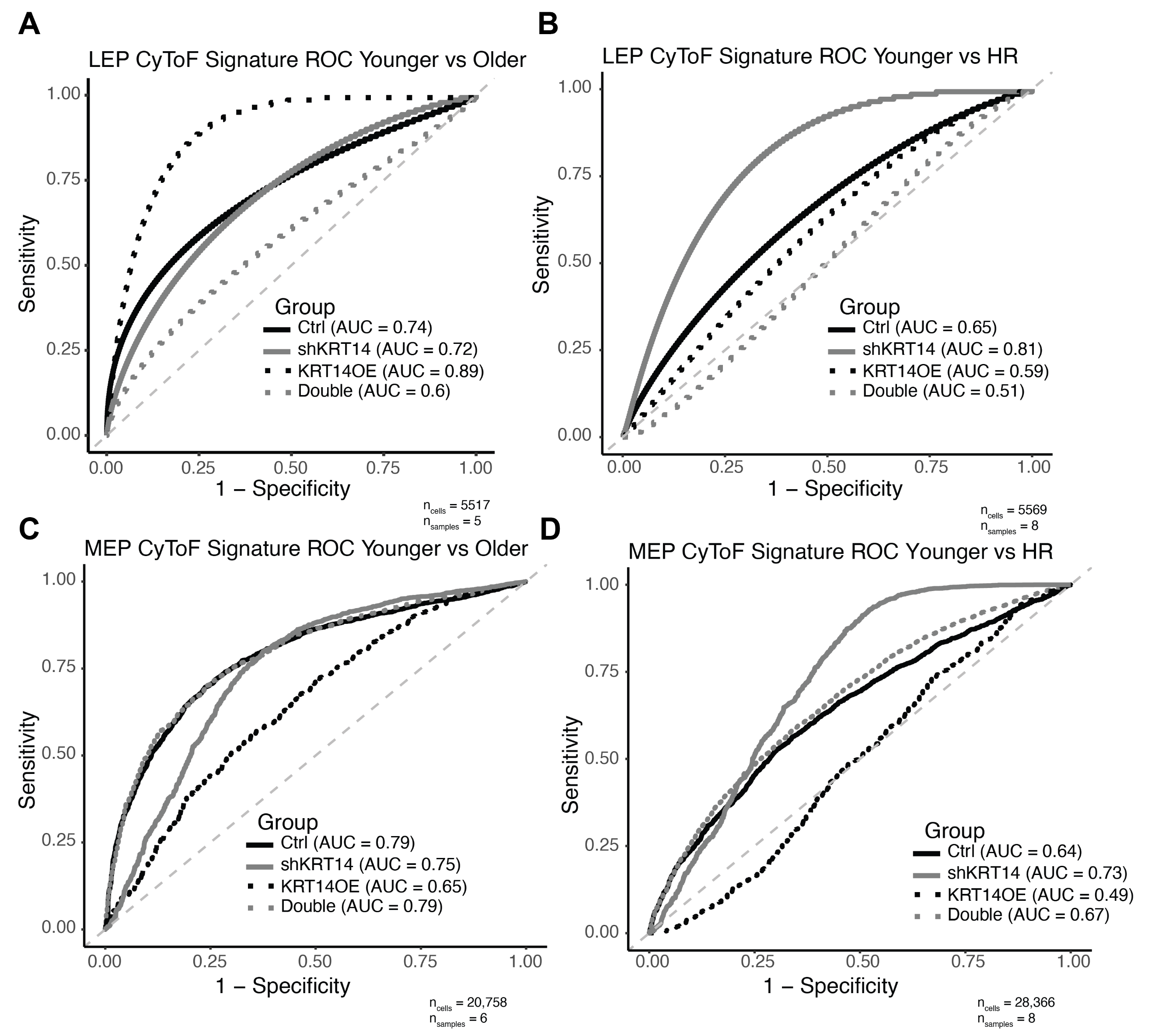


**Supplementary Figure 5: CyToF aging signature performance across epithelial lineages.**

ROC curves for classification of younger vs older (A, C) and younger vs high-risk (HR) (B, D) donors using CyToF ageing signatures in luminal epithelial (A, B) and myoepithelial (C, D) cells. Curves are shown for Ctrl, shKRT14, KRT14OE, and Double conditions; AUC values are indicated in the legends.

## Supplemental Tables

**Supplementary Table 1: Specimen Table**

| Figure | Subfigure | Strain Table |
| --- | --- | --- |
| Figure 1 | E + G  Model Strains | \| Strain \| Age Category \| Chronological Age \| Source \| \| --- \| --- \| --- \| --- \| \| 51L \| younger \| 27 \| RM \| \| 123 \| younger \| 27 \| RM \| \| 124 \| younger \| 29 \| RM \| \| 153 \| older \| 60 \| RM \| \| 160 \| younger \| 16 \| RM \| \| 163 \| younger \| 27 \| RM \| \| 164 \| younger \| 28 \| RM \| \| 168L \| younger \| 19 \| RM \| \| 184 \| younger \| 21 \| RM \| \| 172L \| younger \| 28 \| RM \| \| 178R \| older \| 51 \| RM \| \| 191L \| older \| 56 \| RM \| \| 195L \| younger \| 24 \| RM \| \| 237 \| older \| 66 \| RM \| \| 240L \| younger \| 19 \| RM \| \| 262L \| older \| 66 \| RM \| \| 429ER \| older \| 72 \| RM \| \| 96R \| older \| 66 \| RM \| |
|  | F | \| Strain \| Age Category \| Chronological Age \| Source \| \| --- \| --- \| --- \| --- \| \| 48 \| younger \| 16 \| RM \| \| 97 \| younger \| 28 \| RM \| \| 178 \| older \| 51 \| RM \| \| 169 \| younger \| 24 \| RM \| \| 211C \| older \| 64 \| RM \| |
|  | H + I | \| Label \| Strain \| Risk \| Chronological Age \| Source \| \| --- \| --- \| --- \| --- \| --- \| \| HR-1 \| C003R2 \| BRCA2 \| 33 \| M \| \| HR-2 \| C023SCR \| BRCA1 \| 35 \| M \| \| HR-3 \| C003L \| BRCA2 \| 33 \| M \| \| HR-4 \| C006 \| IDC CL \| 45 \| M \| \| HR-5 \| C007CR \| IDC CL \| 41 \| M \| \| HR-6 \| C010CDR \| DCIS CL \| 45 \| M \| \| HR-7 \| C018CDR \| IBC \| 41 \| M \| \| HR-8 \| C019 \| DCIS CL \| 49 \| M \| \| HR-9 \| C025 \| IBC CL \| 45 \| M \| \| HR-10 \| C028CL \| IBC CL \| 41 \| M \| \| HR-11 \| C032CL \| IDC CL \| 47 \| M \| \| HR-12 \| C048CDL \| DCIS CL \| 43 \| M \| \| HR-13 \| C078SCR \| BRCA1 \| 24 \| M \| |
|  | J + K | \| Label \| Strain \| Age Category \| Chronological Age \| Source \| \| --- \| --- \| --- \| --- \| --- \| \| FHx-1 \| C17L \| younger \| 36 \| M \| \| FHx-2 \| C73CDR \| younger \| 33 \| M \| \| FHx-3 \| C51CDR \| younger \| 33 \| M \| |
| Figure 3 | A + B | \| Label \| Strain \| Age Category \| Chronological Age \| Source \| \| --- \| --- \| --- \| --- \| --- \| \| AR-1 \| 59L \| younger \| 23 \| RM \| \| AR-2 \| 160 \| younger \| 16 \| RM \| \| AR-3 \| 240L \| younger \| 19 \| RM \| |
|  | C | \| Label \| Strain \| Age Category \| Chronological Age \| Source \| \| --- \| --- \| --- \| --- \| --- \| \| AR-4 \| 112R \| older \| 61 \| RM \| \| AR-5 \| 237 \| older \| 66 \| RM \| |
| Figure 3 &  Supplemental Figure 1 | A - E | \| Strain \| Risk \| Age Category \| Chronological Age \| Source \| \| --- \| --- \| --- \| --- \| --- \| \| 29 \| Average Risk \|  \|  \|  \| \| 240L \| Average Risk \| younger \| 19 \| RM \| \| 237 \| Average Risk \| older \| 66 \| RM \| \| 163 \| Average Risk \| younger \| 27 \| RM \| \| 51L \| Average Risk \| younger \| 27 \| RM \| \| 112R \| Average Risk \| older \| 61 \| RM \| \| C063CR \| High Risk \| younger \| 63 \| M \| \| C014R \| High Risk \| older \| 52 \| M \| \| C023 \| High Risk \| younger \| 35 \| M \| \| C046SCL \| High Risk \| older \| 52 \| M \| \| C128R \| High Risk \| older \| 59 \| M \| |

Source: RM = Reduction mammoplasty tissue, M = Mastectomy, IBC = Invasive breast cancer, IDC = Infiltrating ductal carcinoma, CL = contralateral,

**Supplementary Table 2. Applied strain in the contraction channel**

| **Cell type** | | **D_avg_ [µm]** | **s** | **w_c_ [µm]** | **ε_avg_** |
| --- | --- | --- | --- | --- | --- |
| Average Risk | older | 19.2 | 2.5 | 10.5 | 0.45 |
|  | younger | 18.8 | 2.0 | 10.5 | 0.44 |
| High Risk | older | 18.1 | 1.9 | 10.5 | 0.42 |
|  | younger | 17.7 | 2.2 | 10.5 | 0.41 |

D_avg_, s, w_c_, and ε_avg_ correspond to the average free cell diameter (Dcell), cell diameter standard deviation, width of the contraction channel, and ε_avg_ = (D_cell_− w_c_) / D_cell_, respectively.

**Supplementary Table 3: Antibodies for mass cytometry.**

| Element | Mass | Target | Host | Clone | Vendor | Cat# | Lot# | RRID |
| --- | --- | --- | --- | --- | --- | --- | --- | --- |
| Nd | 145 | MEK 1/2 | Rabbit | MEK12S217S221-H2 | Thermo Fisher | MA5-28037 | XC3547831 | AB_2745042 |
| Nd | 146 | mTOR | Mouse | A17024A | BioLegend | 610302 | B273519 | AB_2801106 |
| Sm | 147 | pSTAT5 | Mouse | 47 | Fluidigm | 3147012A | 1701901 | AB_2827887 |
| Nd | 148 | K19 | Mouse | BA17 | Thermo Fisher | 14-9898-82 |  | AB_10598673 |
| Sm | 149 | CD271 | Mouse | C40-1457 | Fluidigm | 3149017B | 2181507 | NA |
| Nd | 150 | pRb | Mouse | J112-906 | Fluidigm | 3150013A | 0361504 | NA |
| Sm | 152 | pAkt | Rabbit | D9E | Fluidigm | 3152005A | 2103273-09 | NA |
| Eu | 153 | pSTAT1 | Mouse | 4a | Fluidigm | 3153005A | 1501704 | NA |
| Sm | 154 | Vimentin | Rabbit | D21H3 | Fluidigm | 3154014A | 0591704 | NA |
| Gd | 155 | GSK3 | Rabbit | 19H1L12 | Thermo Fisher | 702230 | 2084110 | AB_2632971 |
| Gd | 156 | p38 | Rabbit | D3F9 | Fluidigm | 3156002A | 2631813 | AB_3661861 |
| Gd | 157 | Axl | Mouse | 108724 | R&D | MAB154 | GCV0821041 | AB_2062558 |
| Gd | 158 | pSTAT3 | Mouse | 4/P-STAT3 | Fluidigm | 3158005A | 0132015 | AB_2811100 |
| Tb | 159 | pMAPKAPK2 | Rabbit | 27B7 | Fluidigm | 3159010A | 1501813 | NA |
| Gd | 160 | CD133 | Mouse | AC133 | Miltenyi Biotec |  | 130-090-422 | AB_244339 |
| Dy | 161 | Ki-67 | Mouse | B56 | Fluidigm | 3161007B | 0641801 | NA |
| Dy | 162 | MST2 |  | Polyclonal | LSBio |  |  | AB_10796893 |
| Dy | 163 | pPEAK | Rabbit | Polyclonal | Milipore | ABT52 | 3439693 | AB_11204873 |
| Dy | 164 | K7 | Mouse | RCK105 | Fluidigm | 3164028D | 2112418-15 | NA |
| Er | 166 | NF-kBp65 | Mouse | K10-895.12.50 | Fluidigm | 3166006A | 1111703 | NA |
| Er | 167 | YAP1 | Mouse | H-9 | santa cruz biotech | sc-271134 |  | AB_10612397 |
| Er | 168 | pSTAT6 | Mouse |  | Fluidigm | 3168012A | 1422005 | NA |
| Yb | 171 | pERK 1/2 | Rabbit | D13.14.4E | Fluidigm | 3171010A | 0762004 | NA |
| Yb | 172 | pS6 | Mouse | N7-548 | Fluidigm | 3172008A | 2104622-07 | NA |
| Yb | 173 | CD44 | Rat | IM7 | Fluidigm | 3150018B |  | NA |
| Yb | 174 | pJNK | Rabbit | Polyclonal | R&D | AF1205 | HCT0620111 | AB_2140857 |
| Yb | 176 | c-Myc | Mouse | 9E10 | Fluidigm | 3176012B | 1841816 | NA |
