# Supplementary figures and images for: MechanoAge, a machine learning platform to identify individuals susceptible to breast cancer based on mechanical properties of single cells
Source: eBioMedicine. 2026 Apr 23;127:106241. doi: 10.1016/j.ebiom.2026.106241 (PMC13174242; doi:10.1016/j.ebiom.2026.106241)

## Supplemental Western Blots


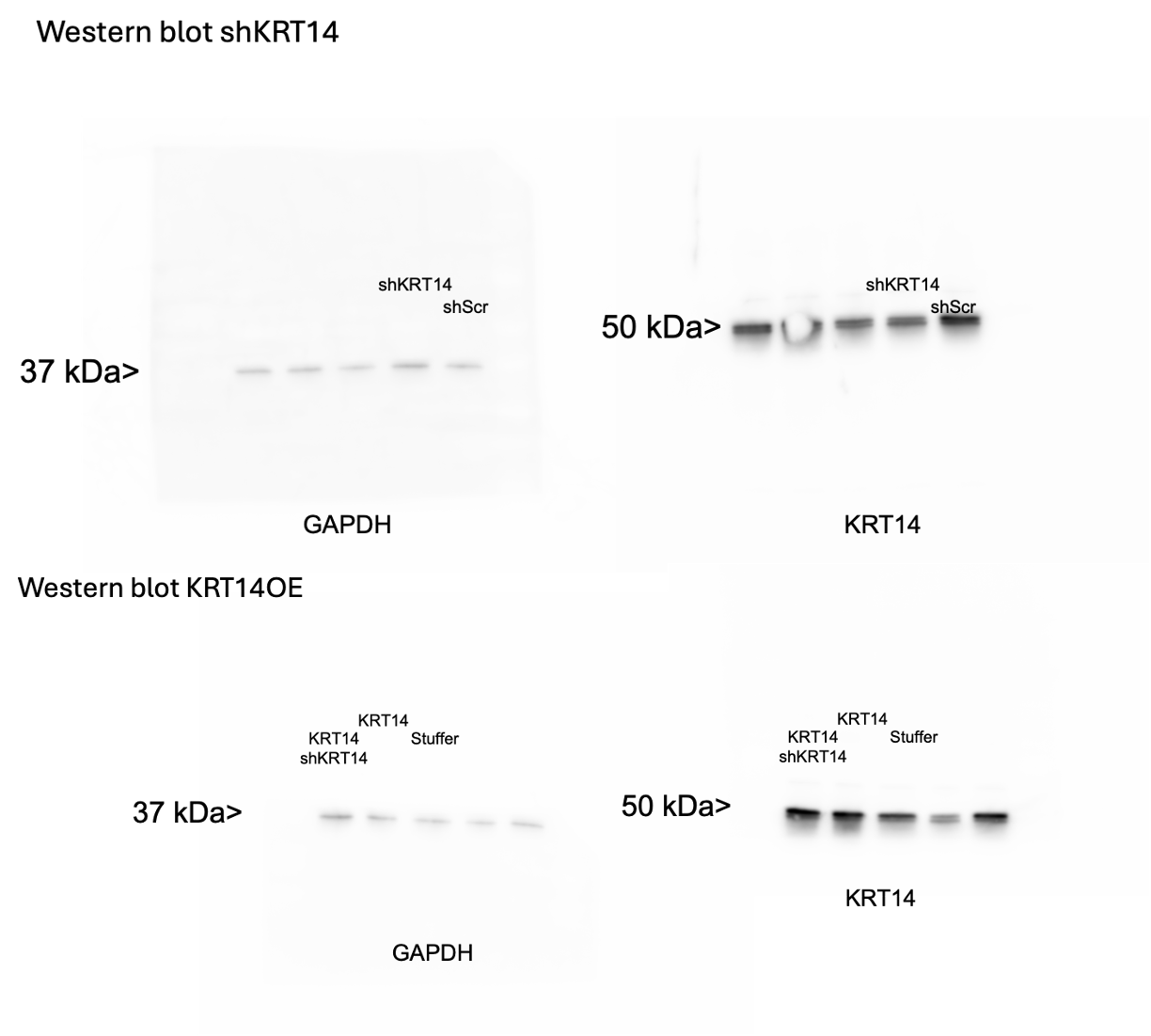

Supplement: Supplemental Western Blots [file mmc2.docx]
